# ICPMS/MS with Benzene Vapor

**DOI:** 10.1021/acs.analchem.4c06171

**Published:** 2025-03-14

**Authors:** Bodo Hattendorf, Tiphanie Renevey, Detlef Günther

**Affiliations:** ETH Zurich, Laboratory for Inorganic Chemistry, Vladimir Prelog Weg 1, CH-8093 Zurich, Switzerland

## Abstract

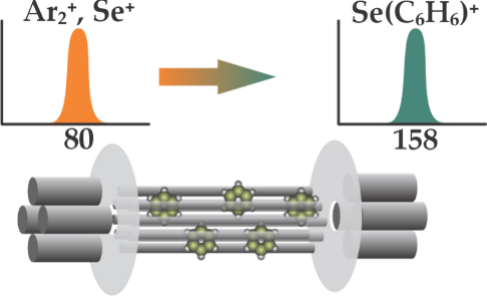

Benzene vapor was introduced into the reaction cell of an inductively coupled plasma tandem mass spectrometer. By evaporating benzene into the helium supply of the octopole reaction cell, product ion spectra for plasma-based atomic and molecular ions were recorded. Based on these spectra, product ions for the separation of the atomic ions from spectral overlaps from isobaric or molecular ion interferents were selected. Background equivalent concentrations (BECs) or sensitivity ratios for analytes and interferents were compared to on-mass analyses without benzene addition. Depending on the analyte, up to 4 orders of magnitude improvement could be achieved. Specifically, the detection of S and Se could be improved substantially, and their BECs were reduced to the μg/L and ng/L ranges, respectively. The separation of isobaric Rb and Sr isotopes or of CeO^+^ from Gd^+^ was less effective with the use of benzene adducts alone. The separation could be substantially improved by using benzene and oxo or water adducts, and analyte/interferent sensitivity ratios greater than 10^4^ were obtained. Finally, the attenuation of ^14^N_2_^+^, interfering with ^28^Si^+^, was evaluated under dry plasma conditions. In this case, benzene could be used to lower the BEC for Si in both on-mass and mass-shift measurements by nearly 3 orders of magnitude.

## Introduction

Gas-phase ion–molecule reactions provide efficient means to overcome spectral overlaps in element and isotope analyses with inductively coupled plasma mass spectrometry (ICPMS).^1^ While sector field instruments allow for separating a wide range of spectral interferences by using a mass spectrometric resolving power up to about 12000,^2^ ion–molecule reactions rely on selective chemical reactions of the respective analyte or interfering ion.^3^ Commercial ICPMS instruments initially utilized the arrangement proposed by Rowan and Houk,^4^ installing a reaction chamber before a quadrupole mass filter. The reaction chamber could be filled with a gas of choice and held a multipole ion guide to mitigate the ion loss caused by scattering. By choosing suitable operating conditions, various spectral overlaps could be resolved by attenuation of interfering plasma background ions such as ArO^+^ or Ar_2_^+^.^4^ About a decade later, commercial instruments successfully utilized different reaction or collision gases to improve the accuracy and precision of quantitative element and isotope analyses. Instruments using hexapole or octopole ion guides, however, were restricted to hydrogen and helium as reaction or collision gases to avoid additional spectral overlaps from complex molecular ions that could be formed inside the reaction chamber.^5,6^ Attenuation of these species could be achieved by so-called energy discrimination methods, where the analyzer quadrupole needed to be set to a more positive potential to suppress the transfer of cell-generated ions of lower axial kinetic energy.^5,7^ The use of a quadrupole with tunable bandpass transmission, on the other hand, allowed better control of the composition of the ion beam inside the reaction chamber, and thus more reactive gases, like ammonia or oxygen, could be used.^8^ Thereby, for the first time, also so-called mass-shift reactions could be employed that make use of reactions in which the isotope of interest reacts with the gas at a much higher rate than the interferent and is detected as the reaction product ion.^9,10^ Nevertheless, in order to maintain transmission toward the mass-analyzing quadrupole, the mass-to-charge (*m*/*z*) bandpass of the ion guide needed to be sufficiently wide to ensure stable trajectories for both the precursor and the product ions, which also led to unwanted reactions, forming new spectral overlaps.^7^

Based on this concept, and initially already suggested by Douglas et al. in 1989,^11^ ICPMS with tandem mass spectrometry (MS/MS) capabilities entered the field.^12^ Using an additional mass-analyzing quadrupole filter before the reaction chamber allows for controlling the composition of the ion beam to a greater degree. Thereby only *m*/*z* ranges of typically 1 amu width are allowed to enter the reaction chamber, providing an unsurpassed control over the reactions that can occur. As the ion population contains only the analyte and interferent of the same nominal *m*/*z*, it is more likely to find a reaction gas that selectively reacts with either the analyte or the interferent. “Selectivity” in this context means that reaction rates of the analyte and interferent(s) under the conditions prevailing in the reaction chamber are sufficiently different so that an unambiguous differentiation in the product ion spectrum is possible. To what extent the reaction rates need to differ is, as such, largely dependent on the relative abundances of analyte and interferent ions in the primary ion beam. Principally, it is, of course, ideal if the reaction rates differ by several orders of magnitude to cover as broad a range of interferent/analyte concentration ratios as possible.

The MS/MS approach allowed for a wider range of reactive gases and reaction pathways with a higher specificity than was previously possible with collision or reaction cells. Apart from helium and hydrogen, where established methods could be transferred from single-quadrupole instruments, now also highly reactive gases could be used for reaction cells equipped with non-mass-resolving ion guides. Most applications to date have used oxygen, but also ammonia and nitrous oxide have been applied to overcome spectral overlaps.^13−15^ Methyl fluoride was used for separation of ^87^Sr^+^ from ^87^Rb^+^,^10,16^ and carbon dioxide and carbon disulfide were employed in fundamental investigations, too.^17,18^

Here, we report on the use of benzene (C_6_H_6_) as a reactive compound in ICPMS/MS applications. Using selected ion flow tube (SIFT) measurements, Koyanagi et al.^19^ studied ion–molecule reactions of benzene with 45 different elemental ions. They showed that benzene reacts with almost all of them at a near collision rate, with exceptions mostly being alkaline and earth-alkaline elements. Its ionization energy (9.25 eV), furthermore, is sufficiently low to allow for charge-exchange reactions with argon and Ar-based molecular ions, although benzene adducts (and eventually bis adducts) appeared to be favored with most elements investigated by Koyanagi et al. Addition of benzene fragments or two benzene molecules was observed for several transition metals, as well. This aspect led us to investigate to what extent benzene may be useful also in ICPMS/MS applications. The caveat of benzene in this context is that its vapor pressure at room temperature is only about 10^4^ Pa, which limits the available concentration in pressurized gas cylinders. To obtain a higher concentration, liquid benzene was evaporated from the headspace of a temperature-controlled reservoir directly into the He gas supply of the ICPMS/MS instrument.

## Experimental Section

### Samples

All solutions were prepared gravimetrically in cleaned polypropylene vials from single-element stock solutions (Inorganic Ventures, Sigma-Aldrich) in 1% (v/v) sub-boiled HNO_3_. Semiconductor-grade silicon was used for laser ablation experiments. Benzene with a purity of 99.5% (Sigma-Aldrich) was used in the MS/MS experiments.

### ICPMS/MS

Reaction profiles were recorded with an Agilent 8900 ICPMS/MS instrument (Agilent Technologies, USA). Solutions were aspirated via the instrument’s peristaltic pump to a MicroMist pneumatic nebulizer (Glass Expansion, Australia), inserted in a temperature-controlled double-pass spray chamber. The operating conditions were initially optimized in no-gas, single-quadrupole mode by aspirating a solution containing 10 μg/L Y to maximize the atomic ion’s sensitivity while maintaining the YO^+^/Y^+^ intensity ratio below 1%. For optimization of the MS/MS mode, a mixture of benzene (*bz* in the following) in helium was fed to the reaction cell via line 3 with a reaction gas flow setting of 10%. Then the settings for the ion optics were adjusted to maximize the ion signal for the (Y-*bz*)^+^ adduct ion, keeping the sample introduction and plasma operating conditions unchanged. The optimized settings were then used in all ICPMS/MS experiments. The helium supply of the reaction cell was from a 6N compressed gas cylinder (PanGas, Switzerland), further purified via a moisture and oxygen trap (Agilent Technologies, USA).

Reaction profiles of N_2_^+^ and Si^+^ were recorded with laser ablation (LA) sampling using an Analyte Femto laser ablation unit (Teledyne-Cetac, USA) with a wavelength of 257 nm. To increase the plasma background signals from N_2_^+^, nitrogen was fed to the ablation chamber via the system’s third mass flow controller. The operating conditions for ICPMS/MS and LA are listed in Table S 1.

### Benzene Vapor Generation

Benzene was introduced into the reaction chamber by evaporation of liquid benzene (ca. 2.5 g) from a stainless steel reservoir, connected to reaction gas line 3 of the reaction cell with 35 cm long, 1/8 in. diameter stainless steel tubing and a ball valve (Figure S 1). The reservoir was placed inside a water bath in a Dewar, and the temperature was held at 25 °C by use of a thermostat (Ecoline RE104, Lauda, Germany) with variation of less than 0.5 °C. The concentration of benzene vapor in helium was estimated from the vapor pressure at the reservoir’s temperature (ca. 100 mbar) and the pressure of the He supply (1.5 bar absolute), resulting in an estimated molar concentration between 6% and 7%. Experiments indicated, however, that the actual concentration of benzene had dropped after the first reaction profile of a series was recorded (Figure S 2), which would imply that the actual concentration was lower than estimated and depended to some extent on the helium flow rate used. Furthermore, we observed that benzene residues may persist inside the instrument for extended periods (Figure S 3).

### Data Evaluation

Reaction profiles for selected product ions were obtained by setting the first quadrupole (Q1) of the MS/MS configuration to the selected precursor ions and recording product ion spectra for *m*/*z* 2–275 via Q2 at increasing flow rates at line 3 of the reaction gas supply. The instrument automatically adds another 1 mL/min of helium via line 1 as soon as line 3 is activated, which was left on throughout all experiments, including the measurements with line 3 set to 0%. The first two spectra were recorded with flow rates set to 0% and 5%, respectively, followed by eight evenly spaced settings until the maximum flow rate. The maximum flow rate had been determined in initial experiments for each precursor ion and corresponded to either the flow at which a background ion signal had dropped to below 10% of the initial ion signal or the point at which the *bz*-adduct of an analyte precursor ion had dropped again by 10% after reaching its maximum. The ion signal intensities for each *m*/*z* were then averaged across all gas flow settings, and an average mass spectrum was plotted to identify the *m*/*z* of abundant reaction products. These were further evaluated by plotting reaction profiles of selected ions, and either the respective background equivalent concentrations (BECs) or the sensitivity ratios were calculated.

## Results and Discussion

### Reaction Pathways

**Figure 1:**
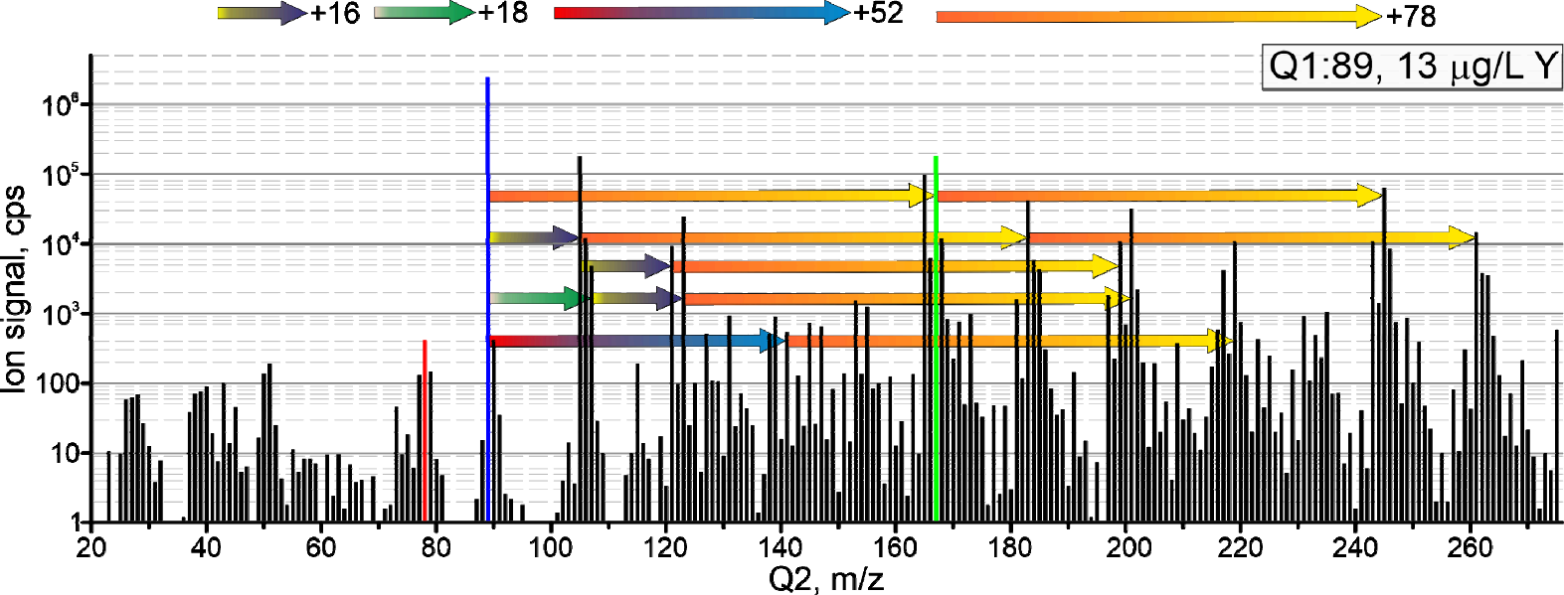
Mean intensities from the reaction profiles of Y^+^, used to identify abundant reaction products. Q1 *m*/*z* is plotted in blue, the most abundant *bz*^+^ isotopologue in red, and reaction products of the target element with benzene providing the highest sensitivity ratio in green. Arrows indicate the mass shifts occurring after addition of benzene (+78), C_4_H_4_ (+52), oxygen (+16), or water (+18) to Y^+^ or its respective reaction products.

The reaction profiles recorded for Y resulted in a variety of product ions that were unexpected. Apart from benzene adduct ions (Y^+^-*bz*_*n*_) (*n* = 1: *m*/*z* 167, *n* = 2: *m*/*z* 245), various additional species were observed (Figure 1). Distinct ion signals were observed at +76 *m*/*z* units, indicating benzyne (C_6_H_4_) addition, which has been observed also for benzene reactions with U^+^, Th^+^,^20^ and Pt^+^.^21^ The adduct ions at +52 *m*/*z* units are considered to be adducts of a C_4_H_4_ fragment of benzene. These adducts had not been reported for Y^+^ to form under thermal conditions when using SIFT experiments,^19^ indicating that the reactions here did not proceed near thermal conditions. The signals appearing at 16 or 18 *m*/*z* units higher than Y^+^ and its benzene adducts are most likely for species containing O and/or H_2_O or formed after benzene addition to YOH_*n*_^+^ (*n* = 0–2). The intensity of YO^+^ formed in the reaction cell, for example, increased continuously with the gas flow rate applied in line 3, while the benzene adduct ions decreased at gas flow rates greater than 2.1 mL/min (see Figure S 4).

### Reactions of Plasma Background Ions with Benzene

**Figure 2:**
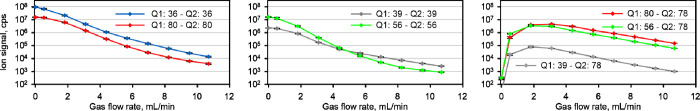
Reaction profiles of plasma background ions with benzene in the reaction chamber. Left: evolution for ^36^Ar^+^ and ^40^Ar_2_^+^; middle: evolution for ^38^ArH^+^ and ^40^ArO^+^, right: profiles representing the respective ionization of benzene. The profile for *bz*^+^ formed by ^36^Ar^+^ is shown in Figure S 2.

Ar-containing plasma background ions reacted by charge exchange to form *bz*^+^ ions and a wealth of species, which are likely from fragmentation of benzene and reactions of fragments with intact molecules inside the reaction cell. Reaction profiles for ^36^Ar^+^, ^40^Ar_2_^+^, ^40^Ar^16^O^+^, and ^38^ArH^+^ with benzene and formation of *bz*^+^ are shown in Figure 2 and Figure S 5. They indicate a rapid charge-transfer reaction with a steep increase of the Q2:78 ion signals. The ultimate decay is likely caused by the formation of adduct ions and scattering losses. The reaction profiles for the parent ions all exhibited a change in their slopes as the flow rates exceeded ca. 5 mL/min, possibly due to dilution of benzene in the gas feed at high gas flow rates.

Benzene fragment ions at *m*/*z* of 52 (^12^C_4_H_4_^+^) and 63 (^12^C_5_H_3_^+^), typical for benzene mass spectra from electron impact ionization,^22^ were especially visible during reaction with ^36^Ar^+^ (Figure S 5). Additional molecular ions occurred in groups near *m*/*z* of 95, 105, 115, 129, 142, 156, 172, 193, 215, 219, and 231 (Figure S 5), which most likely are condensation products of benzene with fragments and/or oxygen species in the reaction chamber. Similar patterns were observed for the product ion spectra of ^40^Ar^16^O^+^ (Figure S 6, top panel) and ^40^Ar_2_^+^ (Figure S 7, top panel). The reaction with ArH^+^ (Figure S 5) did not primarily lead to benzene ionization—ions at *m*/*z* 77 and 79 were present at 2–3 times higher mean intensities. This would indicate that hydride abstraction (<1.8 mL/min) or protonation (>2 mL/min) was the dominant initial reaction channel, according to^23^
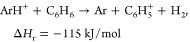

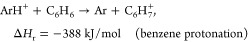


Attenuation of these Ar-based plasma background ions by reaction with benzene was incomplete under these experimental conditions, and only a suppression by up to 4 orders of magnitude was obtained. This limits the use of benzene for on-mass detection of the corresponding interfered analyte ions ^39^K^+^, ^40^Ca^+^, ^56^Fe^+^, and ^80^Se^+^.

### Reaction Profiles for Analyte Ions

**Figure 3:**
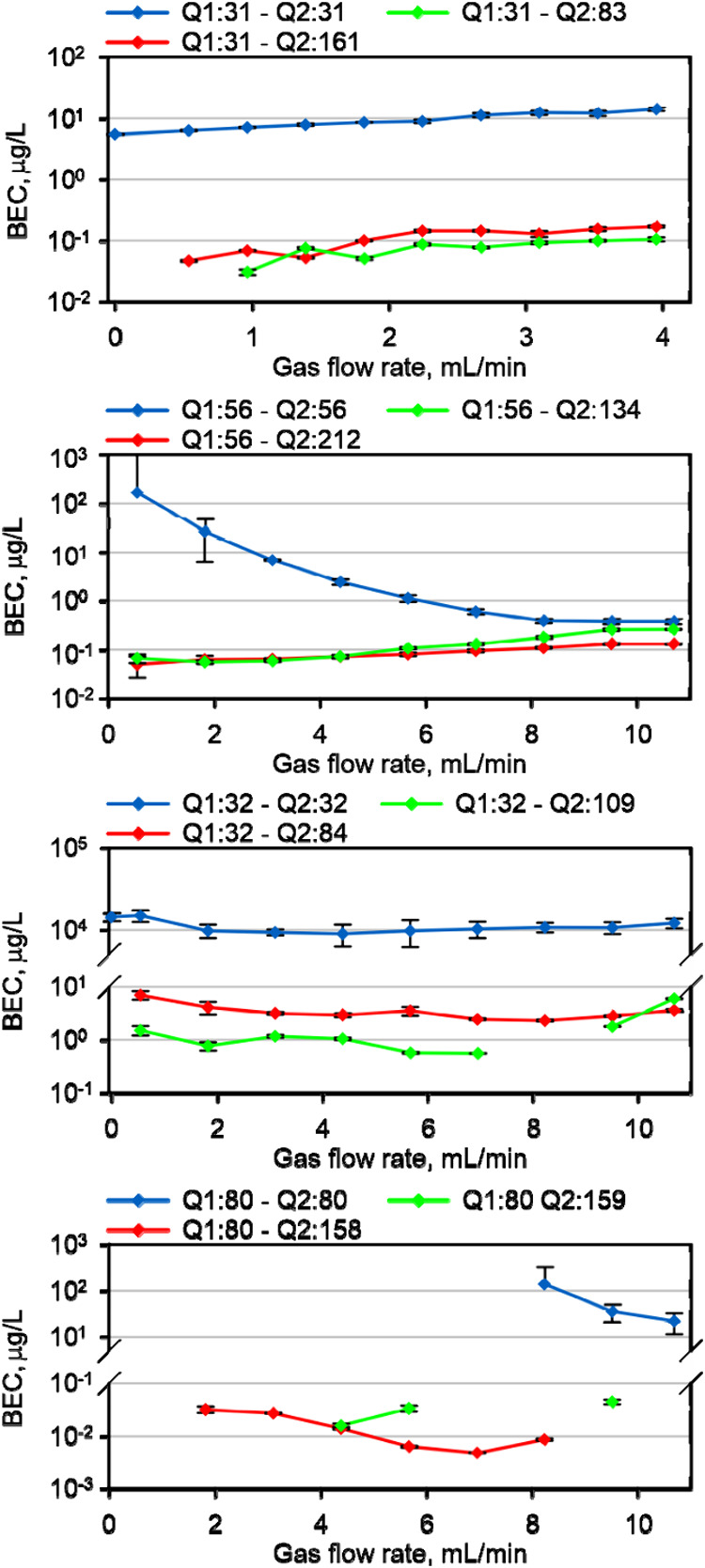
Flow rate dependence of the BECs for P (Q1:31), S (Q1:32), Fe (Q1:56), and Se (Q1:80) from on-mass measurement (Q2=Q1) or using mass shift reactions with benzene. Missing points indicate either indistinguishable ion signals for the blank sample and standard in on-mass mode or zero blank signals for mass shift mode.

K and Ca were reported not to react efficiently with benzene,^19^ which explains why the product ion spectra in the presence of ^40^Ca^+^ were barely distinguishable from those for ^40^Ar^+^ (Figure S 8). Product ion spectra for P, S, Fe, and Se, however, contained adduct ions with benzene and/or its fragments, which were not abundant in the blank spectra. Reaction products of ^31^P^+^ were observed at *m*/*z* 83 and 161 (Figure S 9), which are presumably adducts of the C_4_H_4_ fragment (+52 *m*/*z*) and an additional benzene molecule (+78 *m*/*z*). The BECs of P increased approximately exponentially with gas flow rate, with a similar relative increase for all species (Figure 3). Reaction products reached maximum intensities at flow rates between 1 and 2 mL/min (Figure S 10), where BECs of <100 ng/L were obtained.

Abundant reaction products of S^+^ were also the (S-C_4_H_4_)^+^ adduct at *m*/*z* 84, an ion at *m*/*z* 109—presumably the phenyl (C_6_H_5_) adduct—and a benzene and phenyl adduct at *m*/*z* 187 (Figure S 11). While the intensities for (S-C_4_H_4_)^+^ were approximately 3 times higher, the BECs for the phenyl adduct ion at *m*/*z* 109 were consistently the lowest, with values near 1 μg/L, or 4 orders of magnitude lower than those for on-mass measurements (see Figure 3).

Reactions with Fe^+^ yielded the highest signals for the mono (*m*/*z* 134) and bis (*m*/*z* 212) *bz* adducts (Figure S 6), and the BECs were below 100 ng/L for flow rates between 2 and 5 mL/min, where product ion formation was maximized (Figure S 10). BECs for on-mass measurements approached levels of the mass shift mode at the highest gas flow rates, but sensitivities were then approximately 10 times lower.

The lowest BECs in these experiments were obtained for Se^+^, where the mono adduct (*m*/*z* 158) and an ion at *m*/*z* 159 were detected with high signal/background ratio (Figure S 7). Their BECs were around 10 ng/L, but they showed approximately 10× higher sensitivity at *m*/*z* 158 (Figure S 10).

### Sample-Related Spectral Overlaps

**Figure 4:**
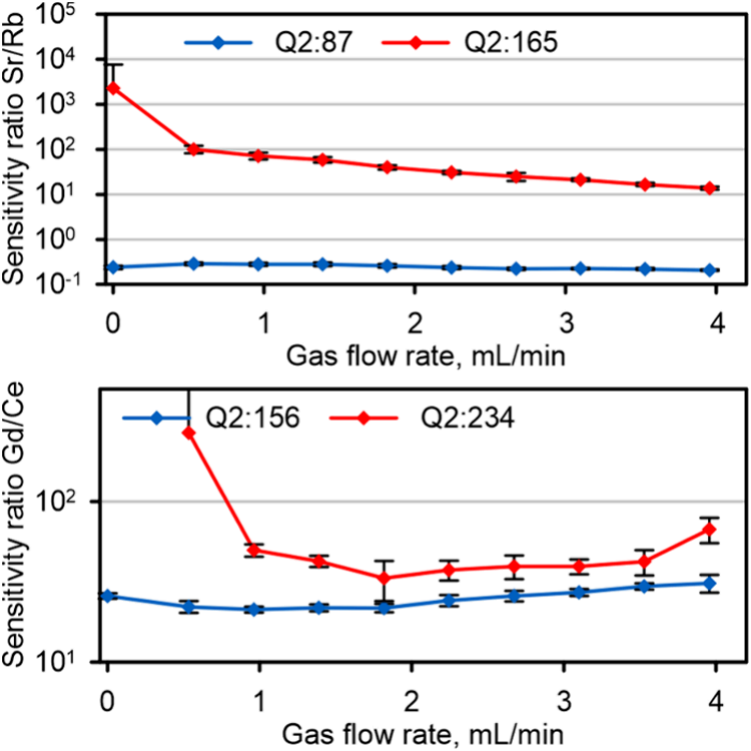
Sensitivity ratios for the on-mass and mass-shifted isotope–interferent pairs Rb–Sr and CeO–Gd.

Spectral overlaps from isobars and oxide molecular ions are among the most difficult to resolve by ICPMS instruments. We evaluated whether there are reaction channels to overcome the interferences from ^87^Rb^+^ on ^87^Sr^+^ and ^140^Ce^16^O^+^ on ^156^Gd^+^. A benzene adduct was observed for Rb^+^ and Sr^+^ but to a much lesser extent for the former (Figure S 12). The sensitivity ratios of the atomic and benzene-only molecular ions at *m*/*z* 165 (Figure 4) indicate that only a moderate separation can be achieved for the isobars. The formation of (Rb-*bz*)^+^ occurred at higher flow rates than that of (Sr-*bz*)^+^ (Figure S 10), but the sensitivity of (Sr-*bz*)^+^ was only about 2 orders of magnitude higher in the best case. Apart from the benzene-only reaction products, a bis oxo adduct ion (*m*/*z* 119) and its benzene adduct (*m*/*z* 197) were observed at high intensity for Sr^+^ but not for Rb^+^ (Figure S 12). The sensitivity ratios for these ions (Figure S 13) allow for detection of Sr without interference even at a 10^5^-fold excess of Rb.

Similarly problematic was the separation of CeO^+^ from Gd^+^ in the mass-shift mode with benzene-only reaction products. Both ions form a mono adduct at *m*/*z* 234 at similar rates (Figure S 10), whereby the sensitivity ratio (Figure 4) only marginally improves compared to that in on-mass measurements. Also in this case, an oxo-ion (*m*/*z* 172) and its hydrate (*m*/*z* 190) as well as their benzene adducts (*m*/*z* 250 and 268) were formed, while they were barely present for CeO^+^ (Figure S 14). The corresponding Gd/Ce sensitivity ratios reached values of 10^4^ (Figure S 13), which could facilitate a practically interference-free analysis in natural samples.

**Figure 5:**
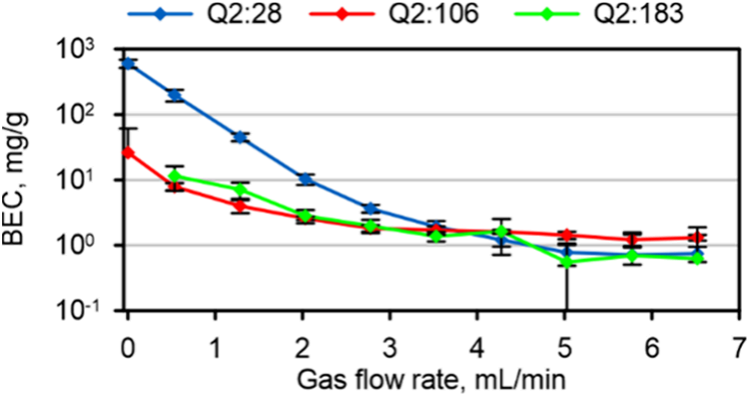
BECs of Si for on-mass (Q2:28) and mass shift analyses (Q2:106, Q2:183) of a Si wafer using laser ablation under dry plasma conditions.

Stimulated by our recent interest in nitrogen-based plasma sources for ICPMS,^24−26^ we were also interested in determining whether benzene can be applied to attenuate the N_2_^+^ interferences of Si^+^. Two abundant product ions at *m*/*z* 106 and *m*/*z* 183 were observed in the average product ion spectra (Figure S 15) for Q1:28, indicating the formation of one benzene and an additional phenyl adduct ion. The reaction profiles for Q2:28, Q2:106, and Q2:183 (Figure S 16) revealed that either on-mass or mass shift measurements may be used to attenuate the N_2_^+^ interference. Product ions at the same *m*/*z* apparently formed more rapidly for the gas blank measurements, reaching maximum intensities at flow rates near 1.5 mL/min, while the product ions during ablation of Si exhibited a broader peak around 2 mL/min. The BECs for all reaction channels, however, approached a very similar value near 1 mg/g at flow rates >3.5 mL/min (Figure 5).

### Summary

Ion–molecule reactions of benzene may be useful for ICPMS/MS applications. Reaction pathways for selected analyte–interferent pairs could be identified, which successfully mitigated spectral overlaps and improved the background equivalent concentrations or sensitivity ratios. Plasma background species, like Ar^+^, ArO^+^, and Ar_2_^+^, were found to yield product ion spectra with far more species than observed, for example, with electron impact ionization. This indicates that the reaction channels available to the ions in an ICPMS/MS instrument are numerous and not all species formed were identified unambiguously. Furthermore, depending on the precursor ion, different main reaction pathways exist, and their product ions can differ substantially, like in reactions of Ar^+^ or ArH^+^. The complexity is partly also reflected in the different most abundant reaction products for the atomic ions observed. While mono and bis adduct ions with benzene occurred in most cases, they did not necessarily constitute the most abundant reaction products. In various cases, product ions containing benzene fragments like C_4_H_4_, phenyl, or benzyne had higher abundances. The product ion yield was, however, in most cases not as high as in SIFT experiments. Nonetheless, with a careful selection of the respective product ion, up to 4 orders of magnitude reduction of the impact of spectral overlap was achieved. The determination of S and Se, in particular, could be improved using mass shifts of 77 and 78 *m*/*z*, respectively, while for Fe (mass shift of 78 *m*/*z*) and P (mass shift of 52 *m*/*z*), BECs only about 3 orders of magnitude lower were obtained. The least improvement with benzene-only reaction products was obtained for the separations of Gd^+^ from CeO^+^ and ^87^Sr^+^ from ^87^Rb^+^. Yet, reaction products containing additional water or oxygen were highly specific, and an efficient separation was achieved in this way. Still, the use of liquid reagents like benzene for ICPMS/MS applications is not straightforward. The approach used in this study—direct evaporation of benzene into the helium gas fed to the reaction cell—affected the reproducibility of the method. Changes in the reaction profiles were obtained, especially after using high helium flow rates, indicating that the effective concentration of benzene was limited by the rate of evaporation and/or diffusion benzene into the gas stream. An optimized approach for controlled addition of compounds of similar volatility, however, can broaden the range of ICPMS/MS applications and help to circumvent spectral overlaps with even higher efficiency.

## Supporting Information Available

ICPMS/MS operating conditions; sketch of the benzene feed to the reaction cell of the instrument; reaction profiles for Y^+^; product ions and repeatability of Y-*bz*^+^ adduct and *bz*^+^ formation; product ion spectra of selected plasma background ions; product ion spectra for ArO^+^ and Fe^+^, Ar_2_^+^ and Se^+^, Ar^+^ and Ca^+^, and NOH^+^ and P^+^; reaction profiles for P, S, Fe, Se, Sr, Rb, CeO, and Gd; product ion spectra for O_2_^+^ and S^+^; sensitivity ratios for reaction products of Sr^+^, Rb^+^, CeO^+^ and Gd^+^, containing not only benzene; and product ion spectra and reaction profiles for N_2_^+^ using dry plasma conditions (PDF)
