# Supplementary material for: ICPMS/MS with Benzene Vapor
Source: Anal Chem. 2025 Mar 14;97(11):5926–31. doi: 10.1021/acs.analchem.4c06171 (PMC11948164; doi:10.1021/acs.analchem.4c06171)
Supplement: Supplementary file 1 — ac4c06171_si_001.pdf [file ac4c06171_si_001.pdf]

Supporting Information
ICPMS/MS with benzene vapor

Bodo Hattendorf, Tiphanie Renevey, Detlef Günther
ETH Zurich, Laboratory for Inorganic Chemistry, Vladimir Prelog Weg 1, CH-8093 Zurich
bodo@inorg.chem.ethz.ch

Table of contents:
Table S1: Operating conditions for the ICPMS/MS and laser ablation systems used.
Stability of the benzene flux.
Figure S 1: Sketch of the experimental setup.
Figure S 2: Repeatability of reaction profiles.
Benzene washout
Figure S 3: Evolution of the ion signal for benzene ionized by $^{40}Ar^+$.
Figure S 4: Reaction Profiles for Y^+.
Figure S 5: Averaged product ion spectra for abundant plasma background ions.
Figure S 6: Averaged product ion spectra for m/z 56.
Figure S 7: Averaged product ion spectra for m/z 80.
Figure S 8: Averaged Product ion spectra for m/z 40
Figure S 9: Averaged product ion spectra for m/z 31
Figure S 10: Reaction profiles for standards
Figure S 11: Averaged product ion spectra for m/z 32.
Figure S 12: Averaged product ion spectra for m/z 87.
Figure S 13: Sensitivity ratios of Sr/Rb and Gd/CeO.
Figure S 14: Averaged product ion spectra for m/z 156.
Figure S 15: Product ion spectra of m/z 28 of a gas blank and LA of a Si wafer.
Figure S 16: Reaction profiles with m/z 28

Table S 1: Operating conditions for the ICPMS/MS and laser ablation systems used.

ICPMS/MS		
RF Power, W	1550	
Sampling Depth, mm	3.0	
Nebulizer Gas, L/min	0.92	
Nebulizer Pump, rps	0.10	
ORS Settings	Initial[a]	Optimized[b]
He Flow Line 1, mL/min	1.0	1.0
He + bz Flow, Line 3, mL/min*	0	0 - 11
Octopole Bias, V	-8.0	3.0
Axial Acceleration, V	1.0	0.0
Energy Discrimination, V	-5.0	-20.0
Laser ablation[c]		
Main Chamber He, L/min		0.150
Inner Cup He, L/min		0.050
Additional Gas N_2, L/min		0.015
Nebulizer Gas (Ar), L/min		0.82
Laser Output, %		15
Repetition Rate, Hz		40
Spot size, μm		10
Scan speed, μm/s		1

a: settings after initial optimization without benzene addition, b: settings for maximum $(Y\text{-bz})^+$ abundance when adding benzene vapor. c: used only for reaction profile of Si.
*: flow rates were converted from software settings in $\%_{FS}$ to ml/min using the manufacturer's recommendations assuming the heat capacity of He gas as: mL/min = $\%_{FS}$ x 0.107, $\%_{FS}$: software setting in % of the mass flow controllers full scale.

Stability of the benzene flux

Figure S 2 shows three consecutive reaction profiles for Y^+ ion signals vs. flow rate of the reaction gas line. Profile 1 was recorded after 30 minutes purging the reaction cell with benzene evaporating (ball valve open) into the helium line 3 at a flow setting of 5% (approx. 0.5 mL/min) and 1 mL/min He via line 1. Recording the reaction profile took approximately 30 minutes and profiles 2 and 3 were recorded in sequence allowing for 6 minutes stabilization in between. After each reaction profile, the flow of line 3 was set to 0% while the reaction cell was purged with 1 mL/min helium from line 1. It can be seen that the first profile exhibits a faster decay of the Y^+ signal (Figure S 2, left) and a steeper increase of the $(Y\text{-}bz)^+$ adduct ion (Figure S 2, middle). The profile for the transition Q1: 36 → Q2: 78 (Figure S 2 right) was additionally recorded in all experiments to monitor ionization of benzene by Ar^+ as an independent indicator for the repeatability of the experimental setup. Also here it was observed that the first profile exhibited a steeper increase of the benzene ion signals compared with the subsequent ones and only became similar as the flow rate exceeded 2.4 mL/min.
Similar differences between the first and subsequent profiles were observed in every experiment after the instrument had been started and warmed up. The difference in the initial profile is considered to be caused by the changes in the benzene concentration during the initial phase of the experiments. Benzene vapor is assumed to have saturated the He supply line during the initial, low flow purge period. Yet, the last

product ion spectrum of a profile's sequence had been recorded with the maximum flow rate (3.1 mL/min in this case), and we assume that the benzene concentration in the supply line was diluted because the evaporation and/or diffusion rate from the reservoir was smaller than what was delivered to the reaction cell. This dilution manifested in a reduced conversion of Y^+, lower abundance of (Y-*bz*)$^+$ adduct ions and less benzene being ionized by $^{36}Ar^+$ in the 2nd and 3rd profiles. The fact that the profiles became more similar at flow rates above 2.4 mL/min would indicate that the evaporation rate of benzene was not sufficient to fully replenish its consumption at this setting already in the first profile. It thus needs to be kept in mind that the set flow rates reported here are not a direct measure for the benzene flux into the reaction chamber, which was a) lower than would be expected from just the vapor pressure of benzene and the pressure in the helium supply and b) most certainly decreasing as the flow rate increased while recording the reaction profile. The general repeatability of the later profiles indicated that at least comparable benzene concentrations were present at a specific flow rate.

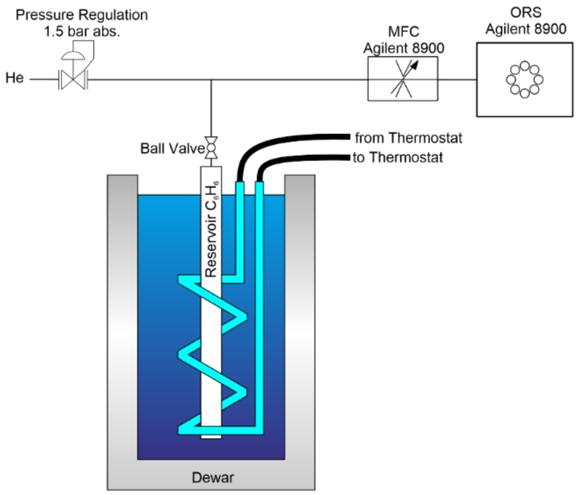

Figure S 1: Sketch of the experimental setup for the introduction of gaseous benzene via the mass flow controller (MFC) into the octopole reaction system (ORS) of the ICPMS/MS instrument

Repeatability of reaction profiles for Y+, Y-bz+ and bz+

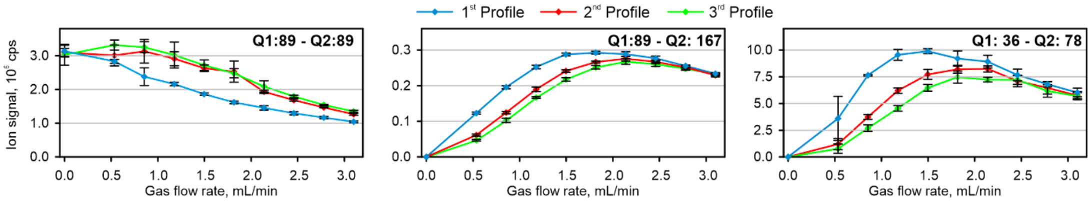

Figure S 2: Consecutive reaction profiles of Y^+ suppression (left), (Y^+-*bz*) formation (middle) and C_6H_6 ionization by $^{36}Ar^+$ (right). The 1st profile was recorded immediately after warm-up and flushing the reaction cell with benzene in He. The 2nd and 3rd profiles were recorded subsequently.

Benzene washout

Benzene residues were found to persist in the instrument for extended periods after the experiments. They are most likely caused by vapor initially adsorbed to the metal surfaces of the gas tubes and mass flow controller. Residual benzene in the reaction cell can most senitively be detected by measuring the intensity of benzene ions (*bz*$^+$) after ionization by $^{40}Ar^+$ via the transition Q1:40-Q2:78. When shutting off the gas supply carrying benzene via the mass flow controller inside the instrument, the *bz*$^+$ intensity initially drops by > 3 orders of magnitude within the first minute followed by a period of 2 hours during which the signal drops by almost another 2 orders of magnitude (Figure S 3). It should be noted however that the ion signal shown is not a direct measure for the concentration of residual benzene vapor, because the ion yield and transmission to Q2 is also affected by the pressure inside the reaction chamber.

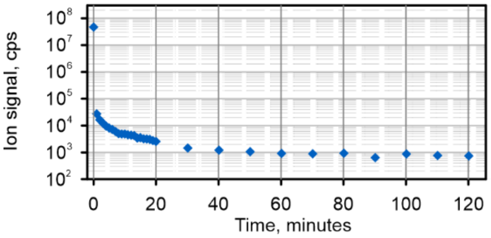

Figure S 3: Evolution of the ion signal for benzene ionized by $^{40}Ar^+$ (monitored via Q1:40-Q2:78). Data were acquired using an initial flow rate of 10.6 mL/min (0 minutes), then stopping the gas supply at line 3 and recording signals at intervals of 1 minute for the first 20 minutes and finally every 10 minutes.

Reaction profiles of Y

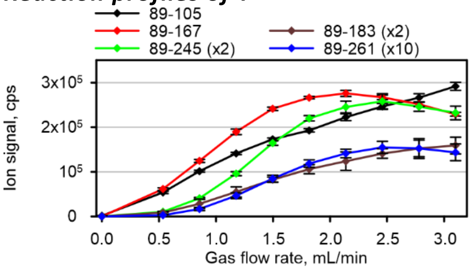

Figure S 4: Reaction profiles of Q1=89 (Y^+) $\rightarrow$ Q2=105 (YO^+), $\rightarrow$Q2= 167 $(Y\text{-}bz)^+$, $\rightarrow$Q2=245 $(Y\text{-}bz_2)^+$, $\rightarrow$Q2=183 $(YO\text{-}bz)^+$, $\rightarrow$Q2= 261 $(YO\text{-}bz_2)^+$.

Product ion spectra of plasma background ions

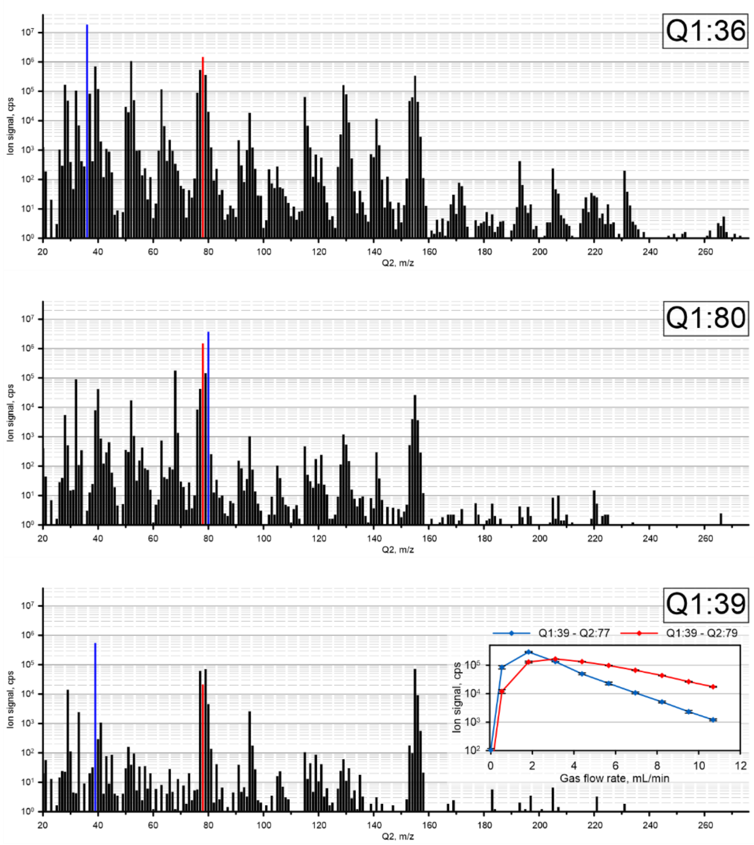

Figure S 5: Averaged product ion spectra for abundant plasma background ions with pneumatic nebulization. Q1 m/z are plotted in blue and bz^+ in red. Insert in the spectrum for Q1:39 shows reaction profiles for Q2 at m/z 77 and 79 respectively. The product ion spectrum for Q1:56 is shown in Figure S 6 top panel.

Product ion spectra for ArO^+ and Fe^+

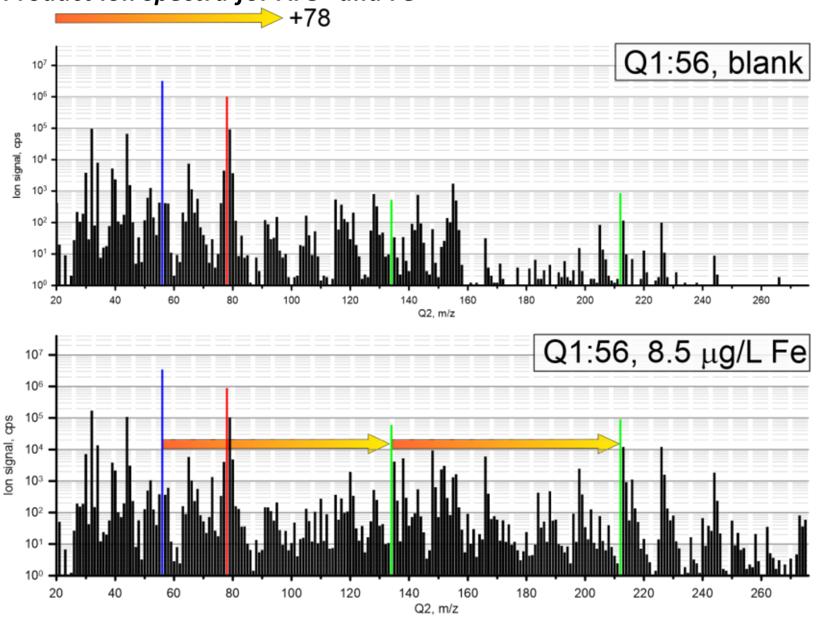

Figure S 6: Averaged product ion spectra for Q1: m/z 56 while aspirating a blank solution and an 8.5 μg/L Fe standard. Q1 m/z is plotted in blue, the most abundant *bz*$^+$ isotopologue in red and reaction products of the target element with benzene providing the lowest BECs in green. Arrows indicate the mass shift by *bz* molecules added.

Product ion spectra for Ar_2^+ and Se^+

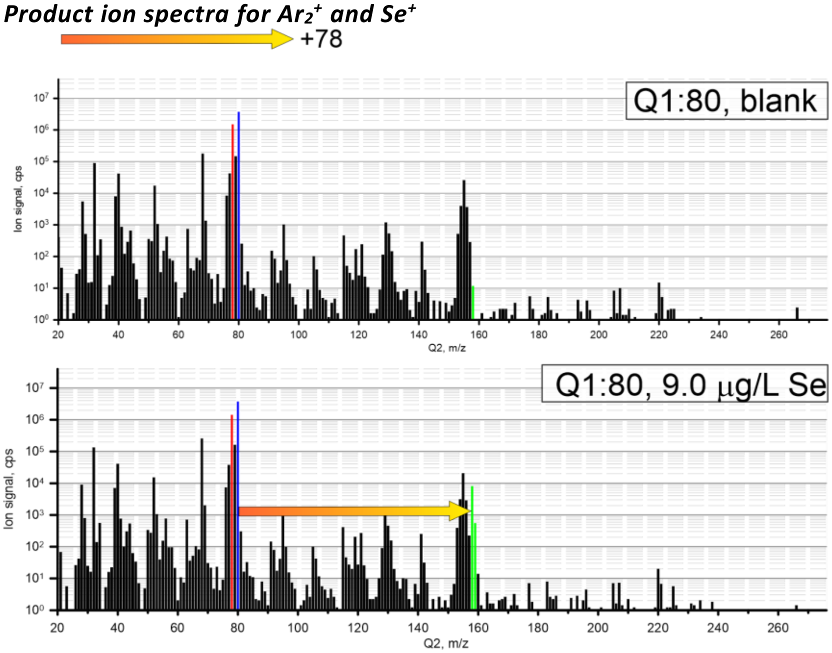

Figure S 7: Averaged product ion spectra for Q1: m/z 80 while aspirating a blank solution and a 9.0 μg/L Se standard. Q1 m/z is plotted in blue, the most abundant *bz*$^+$ isotopologue in red and reaction products of the target element with benzene providing the lowest BECs in green. Arrow indicates the mass shift by *bz* addition.

Product ion spectra for $^{40}Ar^{+}$ and $^{40}Ca^{+}$

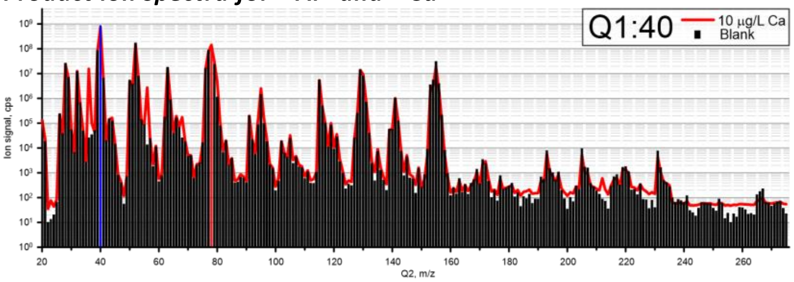

Figure S 8: Averaged Product ion spectra for Q1:40 while aspirating a blank solution and a standard containing Ca. For better visibility the envelope of the spectrum for the standard is shown. Product ions with benzene are not distinguishable. Notable differences are only observed at m/z 36 (not identified) and 56 (probably CaO^{+}).

Product ion spectra for m/z 31

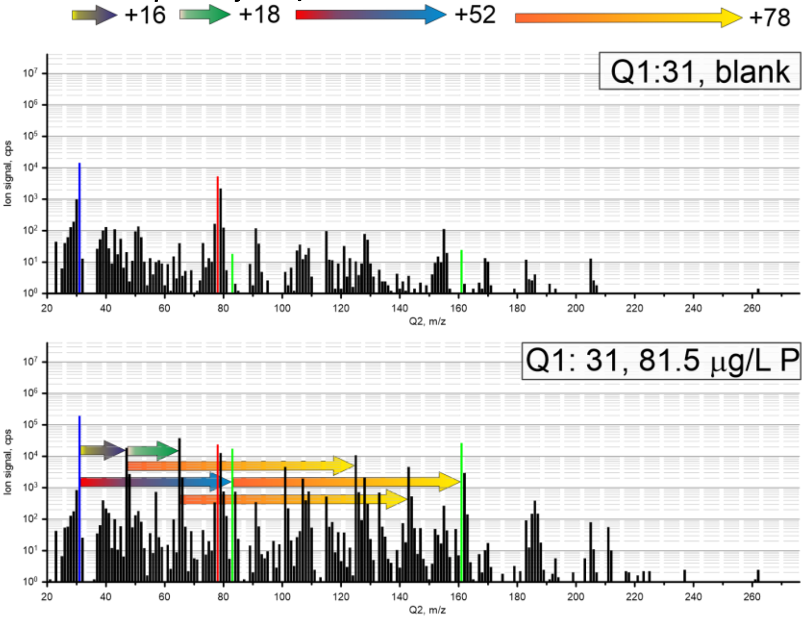

Figure S 9: Averaged product ion spectra for Q1: m/z 31 while aspirating a blank solution and an 81.5 µg/L P standard (top panels). Q1 m/z is plotted in blue, the most abundant *bz*⁺ isotopologue in red and reaction products of the target element with benzene providing the lowest BECs in green. Arrows indicate the mass of the species added to the target isotopes. +78 is most likely intact *bz*, +52 the C_4H_4 fragment, +16 corresponds to oxygen addition and +18 indicates addition of H_2O from impurities in the gas.

Reaction profiles of analyte ions

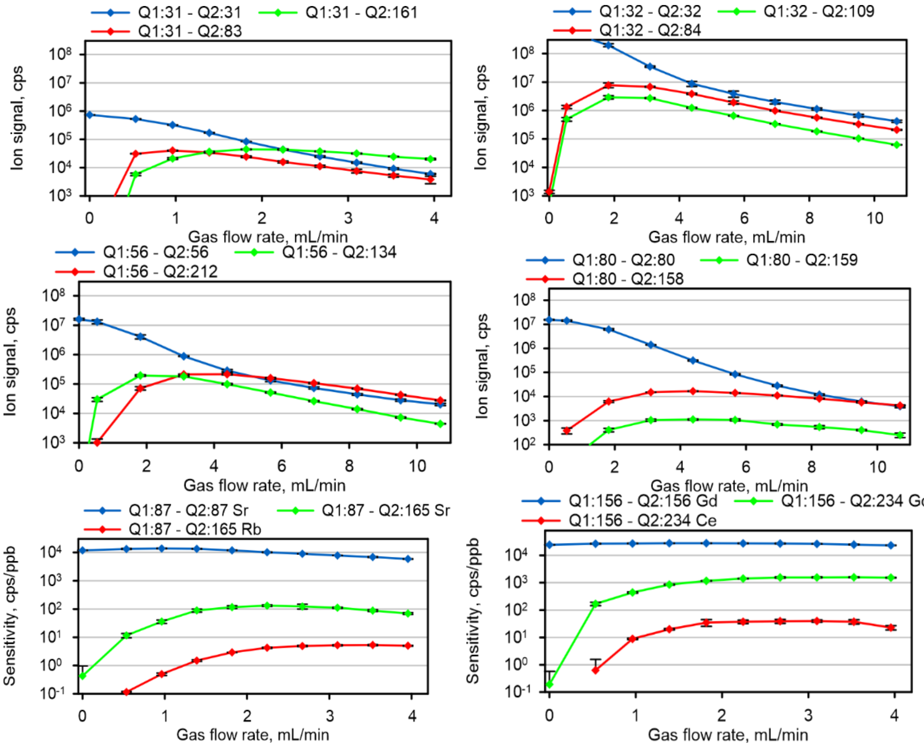

Figure S 10: Reaction profiles for the standard samples of P (Q1:31, 81.5 µg/L P), S (Q1:32, 93.8 mg/L S), Fe (Q1:56, 8.5 µg/L Fe), Se (Q1:80, 9 µg/L Se), Sr (Q1:87, 9.3 µg/L Sr), Rb (Q1:87, 10.3 mg/L Rb), CeO (Q1:156, 9.5 µg/L Ce) and Gd (Q1:156, 10.4 µg/L Gd).

Product ion spectra for analyte ions and blank samples

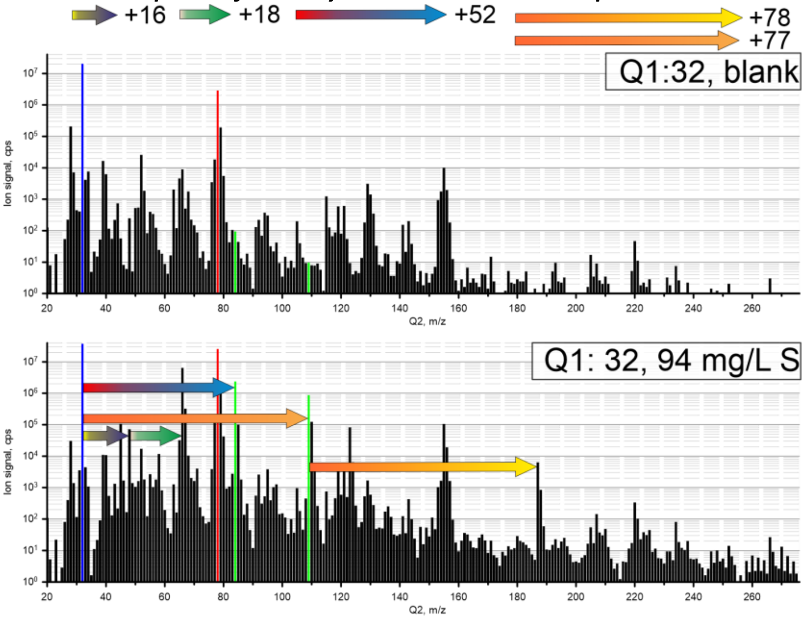

Figure S 11: Averaged product ion spectra for Q1: m/z 32 while aspirating a blank solution and a 94 mg/L S standard (top panels). Q1 m/z is plotted in blue, the most abundant *bz*$^+$ isotopologue in red and reaction products of the target element with benzene providing the lowest BECs in green. Arrows indicate the mass of the species added to the target isotopes.

Interference pairs, Rb^+ - Sr^+ and CeO^+ - Gd^+

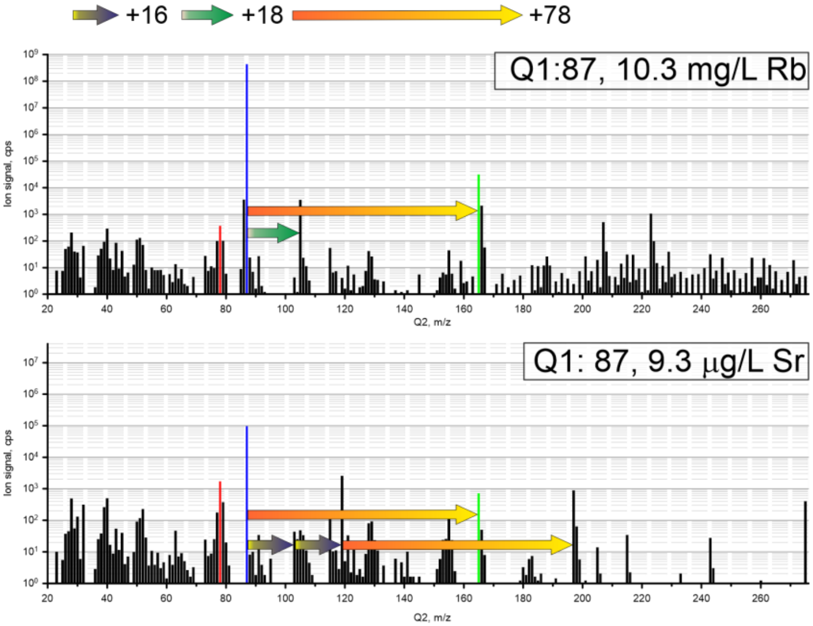

Figure S 12: Averaged product ion spectra for Q1: m/z 87 while aspirating a 10.3 mg/L Rb solution (top panel) or a 9.3 �g/L Sr standard (bottom panel). Q1 m/z is plotted in blue, the most abundant bz^+ isotopologue in red and reaction products of the target element with benzene providing the highest sensitivity ratio in green. Arrows indicate the mass of the species added to the target isotopes.

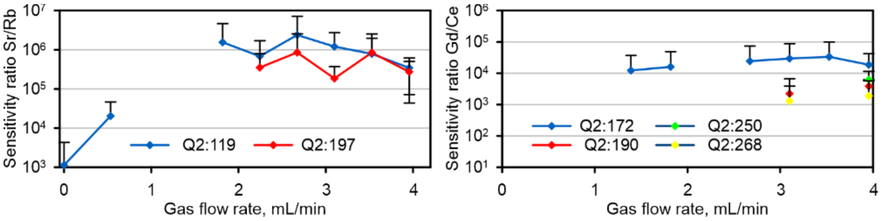

Figure S 13: Sensitivity ratios of Sr/Rb and Gd/CeO for product ions containing not only benzene. Missing points indicate measurements with zero intensities for the interfering isotope.

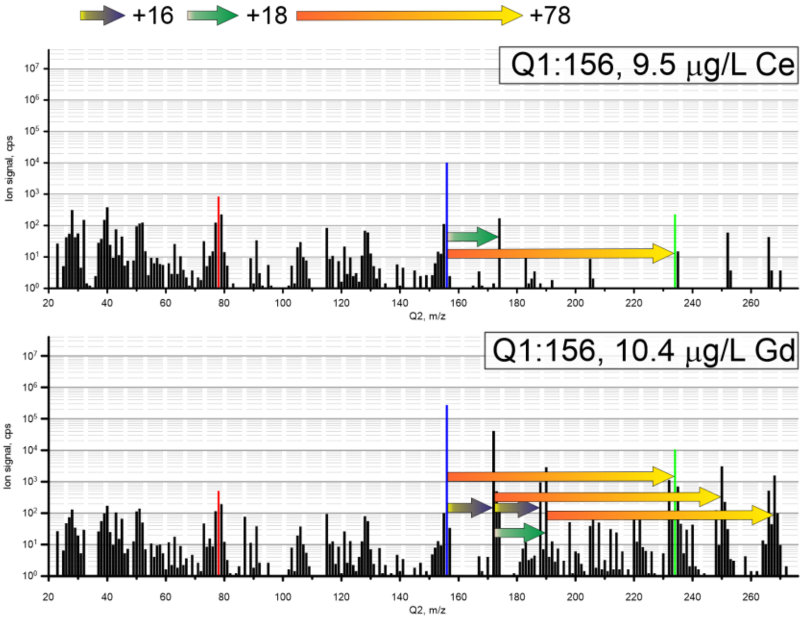

Figure S 14: Averaged product ion spectra for Q1: m/z 156 while aspirating a 9.5 µg/L Ce solution (top) or a 10.4 µg/L Gd standard (bottom). Q1 m/z is plotted in blue, the most abundant bz^+ isotopologue in red and reaction products of the target element with benzene providing the highest sensitivity ratio in green. Arrows indicate the mass of the species added to the target isotopes.

Si vs. N_2, dry plasma

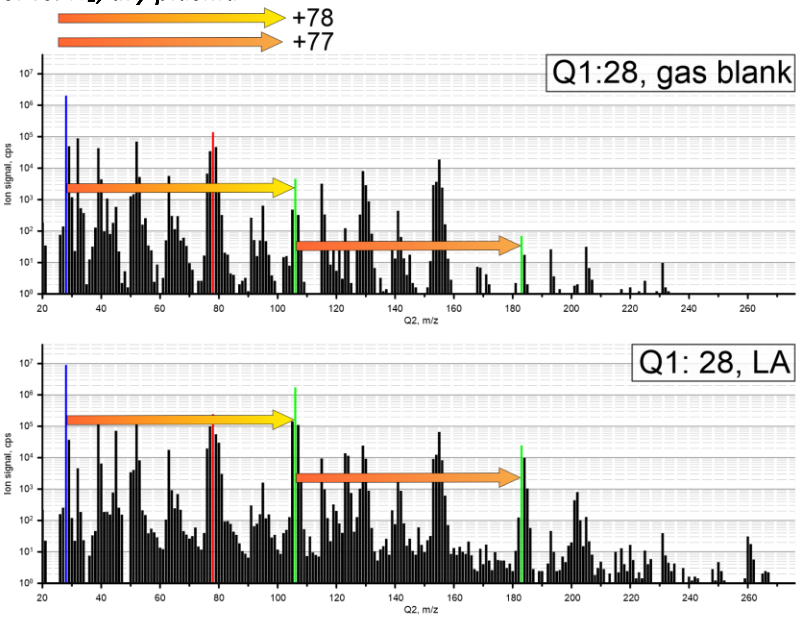

Figure S 15: Product ion spectra of a gas blank and LA of a Si wafer. Q1 m/z is plotted in blue, the most abundant *bz*+ isotopologue in red and reaction products of the target element with benzene providing the highest sensitivity ratio in green. Arrows indicate the mass of the species added to the target isotopes.

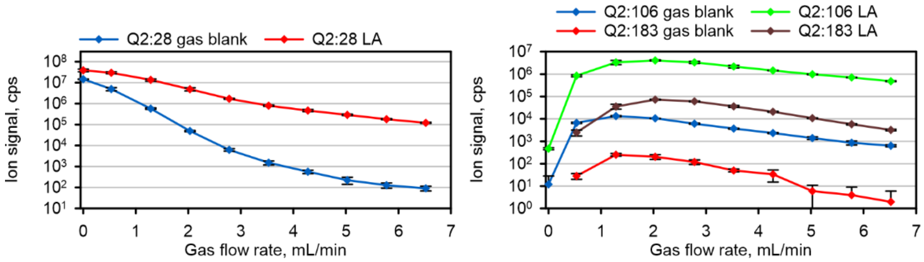

Figure S 16: Reaction profiles with Q1:28 for a gas blank and LA of a Si wafer.
